# Nonenzymatic Glucose Sensors Based on Copper Sulfides: Effect of Binder-Particles Interactions in Drop-Casted Suspensions on Electrodes Electrochemical Performance

**DOI:** 10.3390/s21030802

**Published:** 2021-01-26

**Authors:** Julia Mazurków, Anna Kusior, Marta Radecka

**Affiliations:** Faculty of Materials Science and Ceramics, AGH University of Science and Technology, al. Mickiewicza 30, 30-059 Kraków, Poland; akusior@agh.edu.pl (A.K.); radecka@agh.edu.pl (M.R.)

**Keywords:** glucose sensors, drop casting, electrochemical sensors, copper sulfides, coffee-ring effect, suspension stability

## Abstract

The constant progress in novel nanomaterials synthesis has contributed to the rapid development of nonenzymatic glucose sensors. For working electrodes preparation, drop casting proved to be the most convenient and thus most widely applied method. However, appropriate interpretation of obtained electrochemical signal requires in-depth knowledge of limitations related to this technique. In this study, we prepared solutions based on commonly reported polymers for nanostructures immobilization and investigated their influence on copper sulfides distribution on the electrode. Characterization of suspensions properties and behavior of particles during droplet drying revealed that nonionic polyvinylpyrrolidone (PVP) was favorable for electrodes modification with copper sulfides in comparison with Nafion and chitosan. It ensured homogeneity of the suspension as well as the uniform coverage of the electrode surface with particles, what resulted in increased active surface area and, therefore, higher signal from glucose addition. On the other hand, when cationic chitosan was used as a binder, suspensions were agglomerated and, within dry deposits, a coffee-ring effect was observed. Appropriate adjustment of material and polymer interactions led to enhanced electrode electrochemical performance.

## 1. Introduction

Electrochemical sensors for glucose are up to date the most commercialized and widespread in case of diabetes maintenance. Apart from classical glucometers with disposable test strips, strong emphasis is placed nowadays on systems for continuous glucose monitoring (CGM) [1]. Proposed are implantable devices for highly accurate clinical detection in intravenous blood as well as noninvasive methods for point-of-care monitoring in other biological fluids such as tears, saliva, interstitial fluid or sweat [2,3]. The motivation behind the development of nonenzymatic sensors for above-mentioned CGM systems is the feasibility of direct glucose oxidation on the electrode [4]. The majority of available on-the-market enzymatic sensors relies either on amperometric measurement of consumed oxygen/produced H_2_O_2_ (first generation sensors) or toxic mediators (second generation sensors) [2,5]. Therefore, extensive studies have been carried out on materials which would efficiently substitute enzymes. Among considered materials are noble metals, metal oxides and (less often) sulfides, as well as carbon-based materials [6]. They can play a role of standalone electrodes or be immobilized on the substrate surface, e.g., carbon (CE) or glassy carbon electrode (GCE) [7].

Copper sulfides have been a subject of extensive studies for sensing applications owning to the presence of Cu^2+^/Cu^3+^ redox couple efficiently mediating glucose electrooxidation [8]. Moreover, these compounds are believed to be an economical and promising alternative for enzymes due to their abundance, metal like conductivity and possibility to obtain highly anisotropic structures by varying synthesis conditions [9,10]. Reported have been CuS-based sensors exhibiting exceptional sensitivity, long term stability and short response time [11,12]. The mechanism of glucose detection by copper sulfides is similar to one exhibited by pure copper in alkaline medium; however, higher peak currents can be obtained for these compounds [10]. The first stage of the process involves glucose deprotonation and enediol structure formation, which is subsequently complexed by Cu^2+^. At potential around 0.6 V, Cu^2+^ is oxidized to Cu^3+^. Formation of this highly active species leads to enediol oxidation to gluconolactone, which further undergoes hydrolysis to gluconic acid [10,11].

R. Ahmad et al. review article has divided techniques for nanomaterials deposition on the electrode surface into four main groups, namely coating, direct growth, direct deposition and printing [13]. Among coating methods, drop casting was indicated as the most convenient for preliminary evaluation of material usefulness on the laboratory scale. It consists in suspension or slurry preparation, a drop of which is subsequently transferred onto a cleaned and bare electrode surface and dried. In general, such suspensions comprised a solvent, active material and binder for particles immobilization on the electrode surface [14]. Despite overall simplicity, special attention should be devoted in this technique to suspension properties as well as drying process [7]. In the literature, several factors have been recognized which affect deposited film morphology. The essential point is preparation of homogenous slurry, which is rheologically stable [15]. This can be achieved by adjusting mixing procedure and/or control agents addition [14]. Furthermore, the so-called coffee-ring effect (arising from non-uniform droplet drying) has been reported to facilitate accumulation of particles on the perimeter, where the evaporation rate is typically the highest. Interactions between the particles and surfactants were proved to be crucial in avoiding this effect [16]. Highlighted also has been the influence of particles size and their hardness on coating cracking [17]. Stressed must be that without certain modifications, drop casting is applicable only for small-area electrodes [18].

Among most often applied binders for electrodes preparation using the drop-casting method are the following: Nafion, chitosan and conductive polymers such as polyaniline (PANI), poly(3,4-ethylenedioxythiophene) (PEDOT) or polypyrrole (PPy) [19,20,21]. Only few studies have dealt with polyvinylpyrrolidone (PVP) modified active materials [22,23]. Nafion is a negatively charged ionomer, which possesses hydrophobic tetrafluoroethylene (TFE) backbone and perfluoroalkyl ether (PFAE) side chains terminated with hydrophilic sulfonic acid [24]. High cation conductivity makes it an ideal candidate for fuel cells as permselective membrane [25]. Moreover, biocompatibility, good film forming ability and impermeability to anionic interferences (e.g., ascorbic acid, uric acid, acetaminophen) designate Nafion for biosensors applications [19,26]. Chitosan belongs to the group of cationic polyelectrolytes and is hydrophilic. This polymer is insoluble in water; however, it undergoes protonation in acidic media (pH < 6) and its acetate or hydrochloride can be dissolved. Chitosan is also known from his chelating ability towards metals, e.g., copper, mercury and zinc. It is widely used for biomedical applications because of its biocompatibility, nontoxicity and biodegrability [27]. The literature has reported selectivity of chitosan membranes towards uric and ascorbic acid [28]. PVP consists of hydrophobic alkyl backbone and hydrophilic pyrrolidone moiety as a pendant group [29]. It is soluble in water, highly hygroscopic and nonionic. In liquids solutions, it has exceptional wetting properties and easily forms films [30]. Extensive studies proved that PVP is biological inert, and therefore it is used in pharmacy [31]. Another application field is nanomaterials synthesis, where it can serve as dispersant, surface stabilizer, reductant or growth inductor [32].

This research aims to bring a new insight into the preliminary evaluation of nonenzymatic glucose sensors. However, emphasis was not placed on electrocatalytic properties of active material but secondary factors influencing sensor output. When using the drop-casting technique for electrodes modification, various parameters can be adjusted by the applied binder. Diverse surface chemistry of polymers was recognized as a powerful tool to enhance nonenzymatic materials selectivity based on electrostatic interactions with interfering species [11]. Another important aspect is particles distribution on the electrode surface. Deposits homogeneity has been reported to pose a significant effect on the diffusion of analyte to the geometric area of the electrode [7]. By choosing the appropriate polymer, mitigation of the coffee-ring effect can be achieved [16], which can be beneficial not only for ensuring diffusion linearity but also for electrodes reproducibility [7]. In this work, we provide a systematic study on binder-particles interactions within suspensions and their consequences on obtained deposits patterns. Such approach is a novelty in the field of nonenzymatic glucose sensors. Polymer solutions with different compositions were prepared for active material immobilization on GCE surface using the drop-casting method. Copper sulfides particles behavior within the suspensions as well as drying droplet was evaluated. It was proved that the applied binder plays a crucial role in obtaining stable suspension and uniform coverage of the electrode surface thus influencing voltammetric and amperometric response of the sensor.

## 2. Materials and Methods

### 2.1. Chemicals and Materials

Anhydrous copper chloride (CuCl_2_), thiourea (CH_4_N_2_S), anhydrous ethanol (C_2_H_5_OH), L (+)-ascorbic acid (C_6_H_8_O_6_), anhydrous glucose (C_6_H_12_O_6_), acetic acid (99.5–99.9%, CH_3_COOH), sulfuric acid (95%, H_2_SO_4_) and sodium hydroxide solution (0.1 M, NaOH) were purchased from Avantor, Poland, sucrose (C_12_H_22_O_11_), D-fructose (C_6_H_12_O_6_) and lactic acid solution (88%, C_3_H_6_O_3_) from Chempur, Poland, chitosan (low molecular weight) and Nafion from Sigma-Aldrich, USA, and polyvinylpyrrolidone (M.W. 40,000, PVP) from Alfa Aesar, USA. All chemicals were analytically grade and required no further purification. Glassy carbon electrodes (GCE) with a diameter of 3 mm were obtained from Mineral Company, Poland.

### 2.2. Synthesis of Copper Sulfides

Detailed synthesis procedure is described elsewhere [33]. Briefly, 0.010 mol of CuCl_2_ (1.34 g) and 0.018 mol of thiourea (1.52 g) were dissolved in 160 mL of ethanol. Subsequently, 1 g of PVP was added. Solution was further mixed on the magnetic stirrer for 30 min and transferred to a Teflon-lined stainless-steel autoclave of 300 mL maximum capacity. Temperature was maintained at 200 °C for 6 h. Precipitate was washed with water/ethanol mixture (50%/50% *vol./vol.*) several times and centrifuged at 6000 rpm. Finally, obtained product was dried in a vacuum oven at 60 °C for 12 h.

### 2.3. Characterization of Copper Sulfides

The morphology of obtained nanostructures was characterized using Nova NanoSEM 200 (FEI Company, USA) scanning electron microscope (SEM) and Tecnai TF 20 X-TWIN (FEI Company, Hillsboro, OR, USA) transmission electron microscope (TEM). The crystal structure was determined by the means of X-ray diffraction (XRD) using X’Pert MPD diffractometer (Malvern Panalytical Ltd., UK) equipped with Johansson monochromator (Cu K_α1_ radiation, 1.5406 Å). The scans were collected in the 2θ range of 10–90° with a step size of 0.039°.

### 2.4. Preparation and Characterization of Nanosuspensions

Nanosuspensions for GCE modification were prepared by dissolving certain amount of the polymer (chitosan or PVP) in acetic acid solution with different concentration (C_acid_ = 1%, 5% or 25%). Commercial Nafion resin solution was additionally diluted with deionized water in ratio 1:9 in order to obtain final concentration of 0.5 wt.% and avoid deposited film cracking. To ensure full particles coverage with PVP, its concentration was fixed at 1.0 wt.%. Composition of different nanosuspenions is summarized in Table 1. Next step involved dispersing 0.025 g of copper sulfides in 10 mL of appropriate polymer solution. Suspensions were further homogenized by mixing for 30 min on the magnetic stirrer and then 10 min using ultrasounds.

Viscosity of the polymer solutions was determined using Anton Paar Physica MCR-301 rheometer (Anton Paar GmbH, Austria) equipped with parallel plate PP 25. Gap between disks was set to 0.1 mm. Constant shear rate of 100 s^−1^ and temperature of 22 °C was maintained for 5 min during the measurement. Zeta potential and hydrodynamic diameter of copper sulfides were assessed with electrophoretic light scattering (ELS) and dynamic light scattering (DLS), respectively, by Zetasizer Pro (Malvern Panalytical Ltd., UK) at 25 °C. Zeta potential was measured in deionized water and in PVP-based water solution (m_Cu1.8S/CuS_:m_PVP_ = 1:4 as in PVP_1.0/25%) at different pH values adjusted with 0.01 M HCl.

### 2.5. Preparation of Working Electrodes

Working electrodes were prepared by the drop-casting method. First step involved pre-treatment of glassy carbon electrodes (GCE). Their surface was polished with alumina slurry (0.3 µm) to a mirror-like finish and subsequently cleaned with deionized water and ethanol. After drying, GCEs were activated following the procedure proposed by Z. Gao et al. For this purpose, cleaned GCEs were held in 0.1 M H_2_SO_4_ at +2000 mV for 30 s and at −1000 mV for 10 s. Subsequently, electrodes were scanned from 0 to 1000 mV (scan rate: 100 mV/s) by the means of cyclic voltammetry until voltammograms were reproducible. Finally, 10 µL of adequate nanosuspension was casted on as prepared GCE surface and left for drying overnight. When not in use, electrodes were kept in the refrigerator suspended over the deionized water.

Behavior of particles in drying droplet was investigated by casting 10 µL of prepared suspensions onto watch glass. After solvent evaporation, deposits were observed using Leica DVM6 microscope (Leica Microsystems GmbH, Germany) and captured by LAS X software. Particles distribution on the electrodes surface was closely examined by Phenom XL scanning electron microscope (Thermo Fisher Scientific, USA) at different magnifications.

### 2.6. Electrochemical Measurements

Electrochemical measurements, i.e., cyclic voltammetry (CV) and chronoamperometry (CA), were performed on a M161E electrochemical work station (MTM Anko, Poland) connected to a conventional three electrode cell with the modified GCE electrode as a working electrode (WE), a Pt wire as a counter electrode (AUX) and Ag/AgCl (3.0 M KCl) electrode as a reference electrode (REF). Voltammograms were recorded with the scan rate 100 mV/s in the range of −100 to 1000 mV. The role of electrolyte played 0.1 M KCl + 1 mM K_3_[Fe(CN)_6_] solution in case of active surface area measurements and 0.1 M NaOH for the research including glucose detection. Amperometric response of the modified electrodes to glucose was acquired at fixed potential determined in CV. Before the exact measurement, any air bubbles present on the electrodes surface were carefully removed and 10 scans were performed as preconditioning phase.

Chemical stability of the electrodes after CV measurements in the concentration range 0–1 mM (step 0.1 mM) was investigated by Raman spectroscopy using WITec’s Raman confocal microscope alpha 300R (WITec GmbH, Germany). Spectra were acquired in the range of 200–1600 cm^−1^ with 488 nm excitation wavelength and 100 × objective. Acquisition time was set to 1 s and the number of accumulations to 10.

## 3. Results

### 3.1. Structure and Morphology

**Figure 1:**
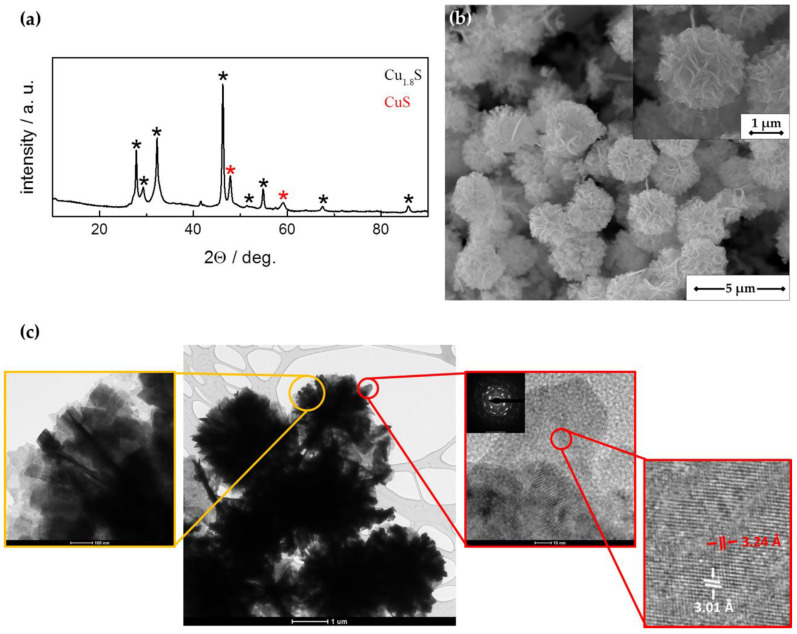
Characterization of copper sulfides: (**a**) X-ray diffraction pattern; (**b**) SEM images with different magnifications; (**c**) TEM and HR-TEM images showing d-spacing 3.24 Å and 3.01 Å corresponding to (101) plane of CuS and (018) plane of Cu_1.8_S, respectively.

The XRD pattern of synthesized material is shown in Figure 1a. Analysis indicated the presence of two copper sulfide phases within the nanostructures: dominant digenite (Cu_1.8_S, R3m) and less pronounced covelline (CuS, P6_3_/mmc space group). Deviation from initial molar ratio of Cu:S = 1:1.8 can be explained by a tendency of copper ions to diffuse in the lattice, leaving the vacancies and taking interstitial positions [34].

SEM examination of material morphology presented in Figure 1b indicates that flower-like nanostructures were obtained. They were well dispersed and 2–3.5 µm in diameter. Detailed morphology and structure studies were carried out using HR-TEM and electron diffraction from the platelet (Figure 1c). It was revealed that copper sulfides nanostructures were composed of nanosheets assembled together during synthesis process. The lattice spacings of 3.01 Å and 3.24 Å corresponding to the d-spacing between (018) crystallographic faces of Cu_1.8_S and (101) facets of CuS, respectively, were observed.

According to DLS measurements in water shown in Figure S1, average hydrodynamic diameter of copper sulfides was 0.9 µm. Nonetheless, particles were characterized by wide size distribution and their size varied in the range of 0.3–5.0 µm.

### 3.2. Nanosuspensions Properties

**Figure 2:**
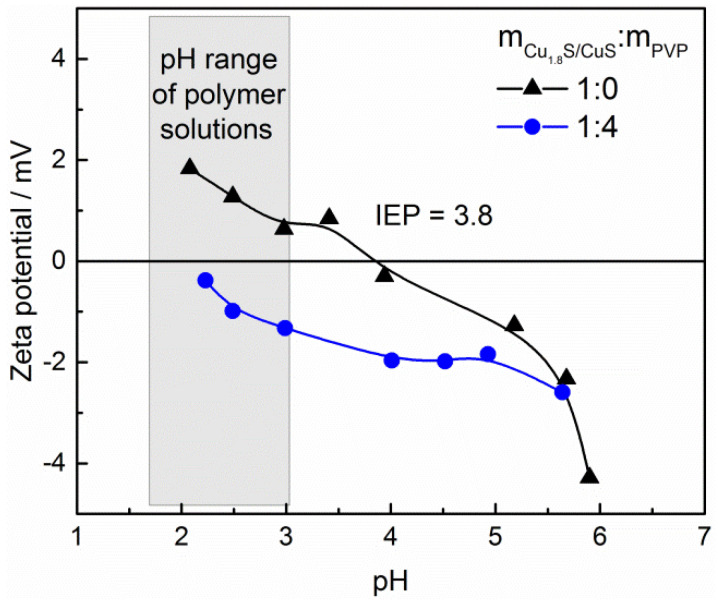
Zeta potential curves for copper sulfides in water and PVP solution (m_Cu1.8S/CuS_:m_PVP_ = 1:4).

For better understanding of nanosuspensions properties, zeta potential dependence on pH was determined for copper sulfides. According to literature data, isoelectric point (IEP) of pure metal sulfides lies close to one for elemental sulfur, i.e., 1.6. However, with increasing oxidation degree, this value can shift towards alkali region and IEP of adequate metal oxide [35,36]. Reported IEPs for CuS are around 1 (precipitation synthesis under constant nitrogen flow) [37] but also as high as 5.8 (hydrothermal synthesis) [38]. Results obtained in this research indicate that IEP of synthesized nanostructures was 3.8 (Figure 2). This value was higher than expected for pure copper sulfides though still far from one characteristic for copper (II) oxide—9.5 [39], what can imply minor degree of surface oxidation. In the presence of PVP (weight ratio m_Cu1.8S/CuS_:m_PVP_ = 1:4 as in PVP_1.0/25% sample), values of zeta potential of copper sulfides were decreased and less differentiated in investigated pH range (Figure 2). These observations are consisted with results obtained in the study of J. Li et al., in which effect of PVP concentration and molecular weight on barium titanate dispersion and size was investigated [40]. Therefore, it can be concluded that proposed polymer content ensured full coverage of particles with the polymer.

Behavior of nanoparticles in polymer solution depends on its pH, viscosity, polymer chain length and presence of functional groups within this chain [41,42]. Molecular weight provided in product specifications of applied polymers was in the range of 50,000 to 190,000 Da for chitosan, for PVP—40,000 Da and for Nafion—100,000 Da. Results obtained from viscosity and pH measurements of prepared polymer-based solutions are summarized in Table 2. It can be clearly seen that pH of chitosan solutions was determined by the acetic acid concentration (pH of 1% acetic acid was measured to be 2.67, 5%–2.26 and 25%–1.72), whereas polymer content had negligible influence. With increasing CH_3_COOH concentration, pH decreased from 2.84 for CH_0.5/1% to 1.85 for CH_0.5/25% and this value was maintained regardless of different polymer additions. It can be assumed that the protonation mechanism of chitosan solubility led to formation of buffer, in which higher dissociation of acetic acid (being in excess) was facilitated by protonation of chitosan. On the other hand, viscosity (*η*) of chitosan solutions strongly depended on its content increasing from 4.85 to 73.12 mPa∙s for CH_0.1/25% and CH_1.0/25%, respectively. Solvent had a minor effect on the *η* value, which was slightly higher in case of concentrated solvents (increase from 14.36 mPa∙s to 21.37 mPa∙s), which was in good agreement with observations of M. Rinaudo et al. [43]. For PVP_1.0/25%, pH was also determined by used solvent, i.e., 25% acetic acid, whereas its viscosity was significantly lower (*η* = 2.03) than one for chitosan solution with adequate polymer content CH_1.0/25% (*η* = 73.12). Due to deprotonation of Nafion in aqueous medium, its pH was within acidic range (2.60). Provided by the supplier, the viscosity of the undiluted Nafion resin solution (5 wt.%) at temperature of 20 °C was in the range of 23–35 mPa∙s, whereas the diluted solution was 1.26 mPa∙s.

As can be seen in Table 2, the hydrodynamic diameter (*D_hyd_*) of copper sulfides in nanosuspenions varied depending on the composition of polymer solution. The highest values were observed in case of increasing polymer load in solution (4.20 µm in CH_1.0/25%) and decreasing acetic acid concentration (4.04 µm in CH_0.5/1%). On the contrary, PVP- and Nafion-based supensions were characterized by lower measured *D_hyd_* of copper sulfides—1.19 and 1.60 µm, respectively.

### 3.3. Drop Casting

**Figure 3:**
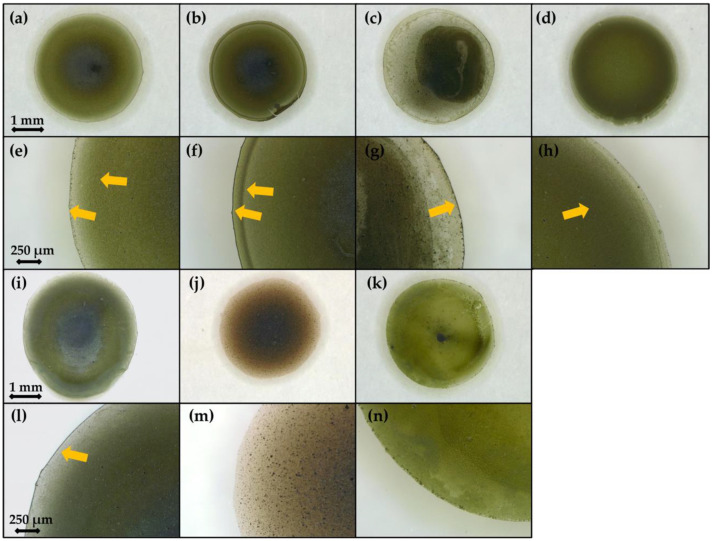
Microscope images of dry deposits: (**a**,**e**) CH_0.5/1%; (**b**,**f**) CH_0.5/5%; (**c**,**g**) CH_0.1/25%; (**d**,**h**) CH_1.0/25%; (**i**,**l**) CH_0.5/25%; (**j**,**m**) NAF_0.5; (**k**,**n**) PVP_1.0/25%. Yellow arrows indicate rings of accumulated particles.

Final particles distribution on electrode surface can vary depending on their behavior in drying droplet [7]. Figure 3 shows microscope images of deposits obtained after solvent evaporation from all described above suspensions. In case of chitosan-based solutions, characteristic ring pattern was observed with dark circle inside (Figure 3a–c,e–g). Only for CH_1.0/25%, ring was broader and no particles were in the center (Figure 3d,f). High Nafion concentration (5 wt.%) led to accumulation of particles majority on the perimeter and film cracking (Figure S2). Therefore, commercial solution was diluted with water in ratio 1:9 as proposed in the literature. Particles within dry deposit of NAF_0.5 thickened towards the middle of the dried droplet. Based on brighter green color of PVP_1.0/25% deposit with small black circle in the middle, it can be assumed that the most uniform particles distribution was received for this sample.

**Figure 4:**
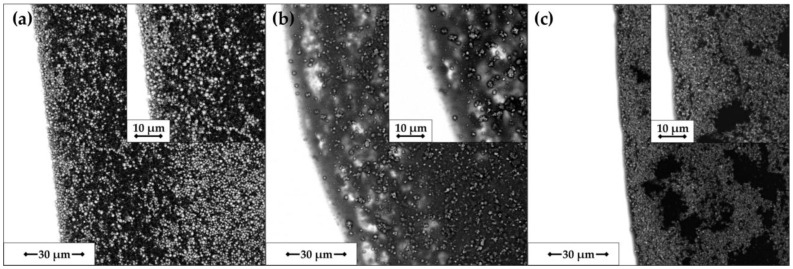
SEM images of dry deposits: (**a**) CH_0.5/25%; (**b**) NAF_0.5; (**c**) PVP_1.0/25%.

In order to gain a better insight into particle distribution on the perimeter of the electrode’s surface, SEM images at different magnifications were collected. As can be seen in Figure 4a, in the case of CH_0.5/25%, a characteristic ring pattern was obtained, followed by loose arrangement and subsequent particle accumulation. For NAF_0.5 (Figure 4b), single, randomly distributed particles were observed at the edge, which thickened towards the center. Sample PVP_1.0/25% was characterized by the most uniform deposit, however, with some voids detected near the perimeter.

### 3.4. Electrochemical Measurements

#### 3.4.1. Effect of the Solvent

**Figure 5:**
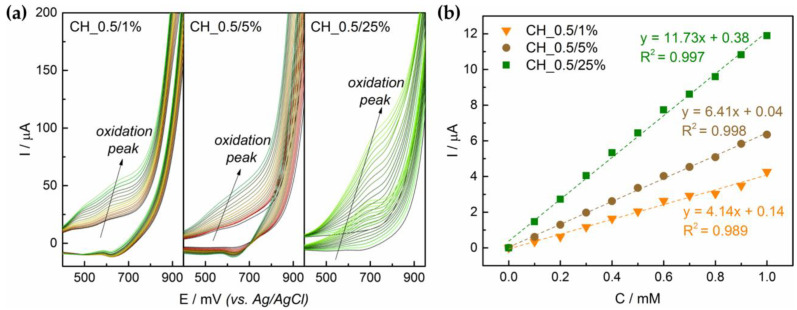
Electrochemical performance of the electrodes based on CH_0.5/1%, CH_0.5/5% and CH_0.5/25% suspensions: (**a**) voltammograms for glucose concentration range 0–1.5 mM; (**b**) regression lines glucose concentration versus generated current (step 0.1 mM).

Results from CV and CA measurements for electrodes based on chitosan solutions with different solvent concentrations are presented in Figure 5. Glucose oxidation potential was determined from the volammograms (Figure 5a). This potential was then applied for amperometric detection. Based on electrodes response to 0.1 mM glucose additions, regression lines current versus concentration were plotted (Figure 5b). For CH_0.5/1% obtained signal per 0.1 mM glucose addition was the smallest, 0.43 ± 0.15 µA. Moreover, calculated for regression line (glucose concentration versus current) coefficient of determination was low (R^2^ = 0.989). Slight increase was recorded for CH_0.5/5%, where the generated current reached a value of 0.64 ± 0.08 µA. In the case of CH_0.5/25%, a more pronounced peak was observed, signal reached the value of 1.17 ± 0.21 µA and a high coefficient of determination (R^2^ = 0.997) was received.

#### 3.4.2. Effect of the Polymer Content

**Figure 6:**
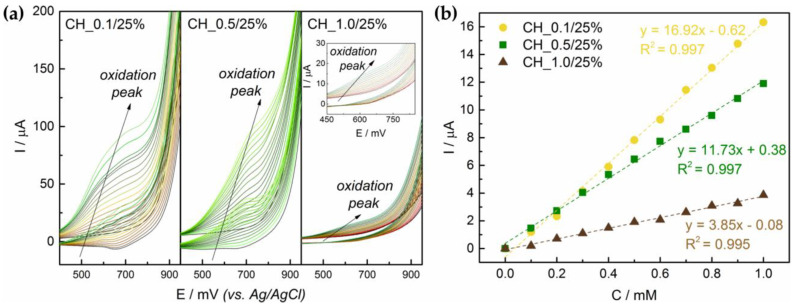
Electrochemical performance of the electrodes based on CH_0.1/25%, CH_0.5/25% and CH_1.0/25% suspensions: (**a**) voltammograms for glucose concentration range 0–1.5 mM; (**b**) regression lines glucose concentration versus generated current (step 0.1 mM).

Polymer content in the suspension proved to have influence on obtained signal from glucose electrooxidation (Figure 6). Response of CH_0.1/25% and CH_0.5/25% to 0.1 mM glucose addition was comparable, 1.63 ± 0.30 µA and 1.17 ± 0.21 µA, respectively, whereas for CH_1.0/25% it was significantly lower at 0.39 ± 0.18 µA. The coefficient of determination did not vary greatly; only slight decrease was observed in case of the electrode with the highest polymer addition (R^2^ = 0.995).

#### 3.4.3. Effect of the Polymer

**Figure 7:**
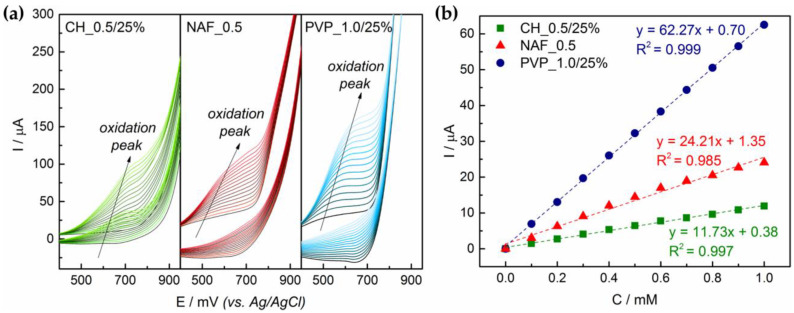
Electrochemical performance of the electrodes based on CH_0.5/25%, CH_0.5/5% and CH_0.5/25% suspensions: (**a**) voltammograms for glucose concentration range 0–1.5 mM; (**b**) regression lines glucose concentration versus generated current (step 0.1 mM).

Comparison between electrodes with different polymers used for copper sulfides attachment revealed that the highest signal was obtained in case of PVP_1.0/25% (Figure 7). For this electrode, a well-defined peak from the electrooxiadtion process was observed, and the generated current per 0.1 mM glucose addition reached the value of 6.23 ± 0.35 µA. Performance of NAF_0.5 was poorer with 2.42 ± 0.94 µA response to the glucose 0.1 mM concentration change. The lowest values were determined for above-mentioned CH_0.5/25% electrode.

Reproducibility of the results was investigated by comparing amperometric responses in the concentration range 0–1.0 mM (step 0.1 mM) of two independently prepared electrodes per sample (see Figure S3). Calculated relative standard error (RSE) was the lowest for PVP_1.0/25% and stood at 1.7%. For Nafion-based electrodes RSE was 4.1% and in case of CH_0.5/25% reached the value of 21.3%. Such high RSE indicates significant sample error. This can be attributed to uncontrolled relative humidity (RH) in the laboratory, which influenced flow patter of particles in nonstabilized suspension during droplet drying. H. Chhasatia et al. reported significant changes in ring pattern formation within colloidal drops deposited on glass substrate at different RH values [44].

Characteristic sensor parameters were evaluated for each of the electrodes to compare their performance. For this purpose, glucose oxidation potential was determined from the first derivative of the voltammograms (Figure S4), whereas limit of detection (*LOD*) and limit of quantification (*LOQ*) were calculated based on regression lines for 0.1 mM additions.

(1) $$LOD=\frac{3.3\;\cdot \;SD}{a},\;$$

(2) $$LOQ=\;\frac{10\;\cdot \;SD}{a},$$

where: *SD* is the standard deviation of blank signal [mM] and *a* is the slope of regression line [µA/mM].

As can be seen in Table 3, determined oxidation potential (E_o_) did not vary significantly between the electrodes, E_o_ = 520 mV for PVP_1.0/25% as well as NAF_0.5 and E_o_ = 540 mV for CH_0.5/25%. Glucose could be detected from 0.002 mM and quantified from 0.005 mM when using PVP_1.0/25%. These values were higher for CH_0.5/25% and NAF_0.5 indicating their inferior analytical properties.

In order to obtain more information about analyzed electrodes, active surface area (*A*) measurements were carried out. For this purpose, voltammograms were recorded with different scan rates (*ν* = 6.25–500 mV/s) in 0.1 M KCl + 1 mM K_3_[Fe(CN)_6_] solution (see Figure S5). Obtained peak currents were plotted versus root of applied scan rate. Using Randles–Sevcik equation, *A* value for each electrode was determined:

(3) $$I=0.4463{\left(\frac{F^{3}}{RT}\right)}^{0.5}An^{5}D^{0.5}C_{0}\nu^{0.5},$$

where: *I* is the peak current [A], *F* is the Faraday constant (*F* = 96,485 C/mol), *R* is the gas constant (*R* = 8.314 J/mol∙K), *T* is the temperature (*T* = 298 K), A is electrochemically active surface area of the electrode (cm^2^), n is the number of electrons involved in the redox reaction (n = 1), *D* is the diffusion coefficient (*D* = 7.2∙10^−6^ cm^2^/s), *C*_0_ is the concentration of K_3_[Fe(CN)_6_] (*C*_0_ = 1 mM) and *ν* is the scan rate [mV/s].

**Figure 8:**
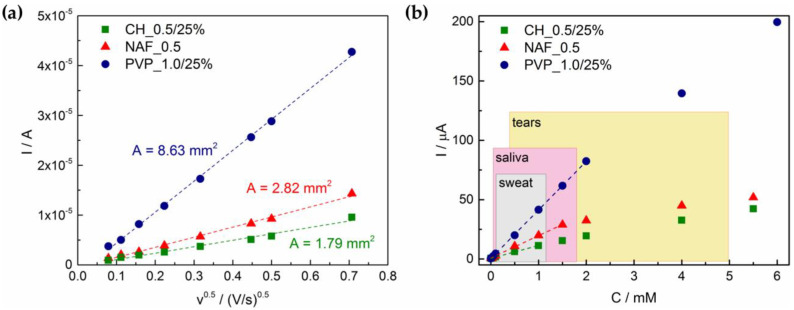
Characterization of the electrodes performance: (**a**) regression lines current versus square root of scan rate; (**b**) calibration plots in the concentration range 0–6 mM with marked linear ranges (dotted lines) and reported glucose levels in aimed biological fluids (boxes) [2].

As can be seen on Figure 8a, regardless of the number of copper sulfides used for nanosuspensions preparation, the highest surface area was detected for PVP_1.0/25%, A = 8.63 mm^2^, whereas for NAF_0.5 and CH_0.5/25% it was 2.82 mm^2^ and 1.79 mm^2^, respectively. As the total surface of unmodified GCE was 7.07 mm^2^, obtained results suggest that in case of Nafion- and chitosan-based electrodes, it was not fully covered with drop casted copper sulfide particles. Only for PVP active surface area increased after modification.

Using values determined from amperometric detection of glucose and active area measurements, sensitivity per area unit (*S*) was calculated using Equation (4):

(4) $$S=\frac{I}{C\;\cdot \;A},$$

where: *I* is the peak current [µA], *C* is the glucose concentration [mM], *A* is the active surface area [cm^2^].

It can be concluded that small changes in glucose concentration influenced the most signal obtained by NAF_0.5 (*S* = 854.29 µA∙mM^−1^∙cm^−2^), whereas slightly weaker effect was observed for PVP- and chitosan-based electrodes, 736.54 and 664.41 µA∙mM^−1^∙cm^−2^, respectively.

The required linear range from sensors varies depending on the aimed biological fluid for analysis. As non-invasive glucose detection methods have recently received significant attention, we aimed to meet the requirements for tears, saliva, or sweat sensors (encountered concentration levels: 0.05–5.00 mM, 0.008–1.77 mM, and 0.01–1.11 mM, respectively [2]). Determined linear range (R^2^ ≥ 0.999) based on calibration curves (Figure 8b) and LOQ values is for CH_0.5/25% between 0.024 and 1.00 mM; for NAF_0.5 between 0.043 and 1.50 mM; and for PVP_1.0/25% between 0.005 and 2.00 mM. These results implicate that PVP-based electrodes can be used in saliva or sweat sensors, whereas CH_0.5/25% and NAF_0.5 did not fully fulfill the requirements for analysis in any biological fluid. Directly recorded amperometric response to changes in glucose concentration are depicted in Figure S6.

Long-term stability is another crucial requirement for glucose sensors. However, its evaluation for drop-casted electrodes is unavailing, considering that deposited suspensions are mostly water-based. During the operation of such electrodes, a binder will tend to redissolve, releasing slowly active material [13]. As copper sulfides are prone to oxidation, the chemical stability of the electrodes was investigated by Raman spectroscopy. For this purpose, spectra of casted nanosuspensions before and after CV measurements (in the concentration range 0–1 mM, step 0.1 mM) were compared. Spectra of pure polymers and copper sulfides were provided for reference. Special attention was devoted to characteristics for copper sulfides pronounced bands at 473 cm^−1^ attributed to S-S stretching modes and less intensive bands at 265 cm^−1^ assigned to Cu-S bonds vibration [45]. Figure S7 shows that for all electrodes, no distinguishable changes in spectra were detected after electrochemical experiments, and the above-mentioned bands were visible at the same positions. The additional broadband near 930 cm^−1^ present in spectra with copper sulfides aroused from the ring vibrations of PVP adsorbed on the synthesis stage [46]. An important aspect to be considered in future studies is conducting more systematic studies after different detection cycles to gain a better insight into copper sulfides’ chemical stability.

## 4. Discussion

**Figure 9:**
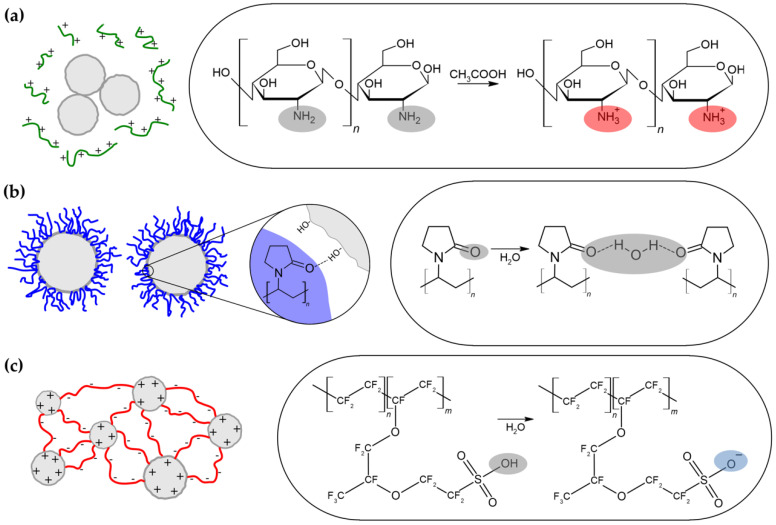
Characteristic suspension stabilization mechanisms exhibited by different polymers: (**a**) chitosan depleted electrostatic stabilization; (**b**) PVP steric stabilization; (**c**) Nafion electrosteric stabilization.

Changes in copper sulfides hydrodynamic diameter in different solutions can be explained by suspension stabilization mechanism exhibited by chosen polymers. Figure 9 presents schematic illustrations of polymers behavior in aqueous solutions and its implication on particles dispersion. As chitosan dissolves in acetic acid, amino groups undergo protonation and subsequently polymer chain gains positive charge [43]. Considering that copper sulfides possess like charge, adsorption of polymer was hindered. Moreover, particles zeta charge, even at low pH (ζ = 1.8 mV at pH = 2.1), was not sufficient to prevent the coagulation (Figure 9a) [47]. Hence, chitosan-based nanosuspensions were characterized by the highest *D_hyd_* of copper sulfides. This value was slightly reduced with increasing acetic acid concentration, because of lower pH of solution and therefore higher *ζ* of particles (electrostatic stabilization). PVP is commonly applied for materials synthesis due to its selective adsorption on certain facets, which leads to the assembly of hierarchical nanostructures. Z. Chen investigated the mechanism of preferential PVP accumulation on Ag (100) planes compared to Ag (111), which contributed to obtaining cubic Ag nanoparticles [48]. Advantage was taken from this characteristic feature of PVP in flower-like copper sulfides synthesis in this study. However, it can be also an explanation of the lowest *D_hyd_* in case of PVP_1.0/25%. Adsorption of polymer on copper sulfides surface prevented particles coagulation in suspensions on the basis of steric stabilization mechanism (Figure 9b). Similar observations were reported by R. Si et al. [49]. During CeO_2_ synthesis, stabilization of cerium nuclei by PVP led to colloid formation. Hydroxyl groups of Nafion can be deprotonated thus leading to negative charge of the chains and electrostatic attraction to positively charged copper sulfides. The same chain can be anchored in two or more particles forming bridges between them. Long Nafion chains ensured sufficient separation (Figure 9c), so *D_hyd_* of copper sulfides in NAF_0.5 was relatively low (electrosteric stabilization). In the study of P.-C. Lee et al., investigated was the influence of interactions between positively charged Pt precursor and the negative sulfonic groups of Nafion on the shape of obtained platinum nanoparticles [50]. However, only the aspect of electrostatic stabilization was brought up.

Suspension homogeneity is not the only factor influencing uniformity of the drop-casted film. Broader perspective on electrochemical performance of the electrodes can be gained by investigating the behavior of particles within sessile droplet. Solvent evaporation imposes certain fall pattern of the particles and when not controlled can lead to their accumulation on the electrode circumference and formation of the so-called coffee-ring effect (CRE). This issue was profoundly addressed by M. Anyfantakis et al. [16]. In their research article, detailed investigation of possible deposits morphology depending on applied ionic surfactant was conducted. It was stated that crucial parameters that needs to be taken into consideration when avoiding CRE are: substrate, particles and surfactant charge as well as surfactant addition and its hydrophobic or hydrophilic character. The most uniform particles distribution was obtained for intermediate surfactant concentration combined with oppositely charged systems, i.e., positively charged particles and anionic surfactant (casted on negatively charged substrate) or negatively charged particles and cationic surfactant (caste on positively charged substrate).

**Figure 10:**
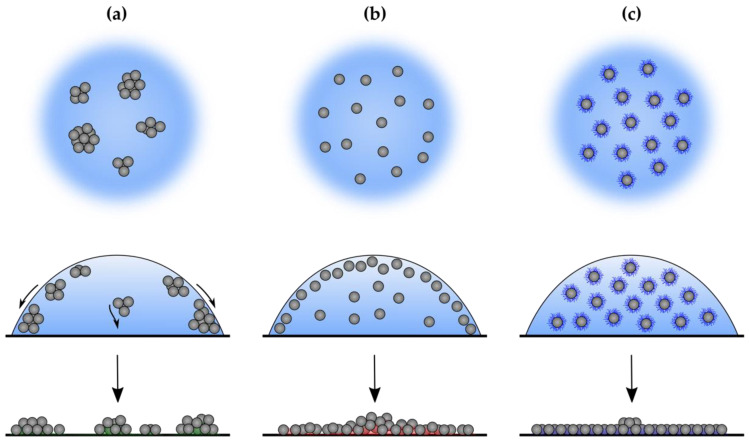
Schematic illustration of particles distribution within the suspension (**top**), sessile droplet (**middle**) and electrode surface (**bottom**) for different binders: (**a**) cationic chitosan; (**b**) anionic Nafion, (**c**) nonionic PVP.

For better explanation of observed CRE in our research and its implication on electrodes response towards glucose, following points must be stated: (1) substrate, i.e., GCE and watch glass, possesses negative surface charge [51]; (2) copper sulfides are positively charged. In case of cationic chitosan, due to its hydrophilic character and negligible adsorption on copper sulfides particles, it was attracted by negatively charged substrate. However, for CH_0.1/25% and CH_0.5/25%, concentration was not sufficient to fully neutralize substrate charge. Therefore, particles were accumulated on the perimeter but also in the middle of the substrate (Figure 10a). For 1.0 wt.% addition, substrate gained positive charge and repulsed like-charged copper sulfides thus promoting capillary-induced flow of particles and formation of CRE. For Nafion, oppositely charged system was formed (Figure 10b). Charge of particles was neutralized and they become hydrophobic owning to tetrafluoroethylene (TFE) backbone of adsorbed polymer. This led to their movement toward liquid-gas interface and entrapment there during droplet drying and meniscus changes. As the copper sulfides were bridged, their concentration in sessile droplet increased towards the center (Figure 9b). Full coverage of particles with nonionic PVP resulted in their amphiphilic character and ensured good dispersion. It also aided in uniform spreading over the substrate (Figure 10c). Role of nonionic surfactants in avoiding CRE was also discussed by Y. Deng et al. [52].

Preferential accumulation of particles on the electrode perimeter and formation of the coffee-ring effect during solvent evaporation significantly altered electrodes response. Observed in CH_1.0/25% broad ring of particles led to severely decreased signal form glucose oxidation. When copper sulfides particles were accumulated also in the middle of the film, signal was higher. In case of NAF_0.5, increasing particles concentration towards the center of the deposit lowered electrode active surface area as well as generated current values. PVP-based solution ensured uniform electrode coverage, leading to the highest active surface area value and obtained response to glucose addition. Broadening of the peak for CH_0.5/25% can be attributed to microelectrodes formation within each aggregate, whereas well-defined peaks in case of PVP-based electrodes were the result of comparatively more uniform distribution of particles on the GCE surface.

## 5. Conclusions

In summary, we investigated the influence of secondary, suspension-related parameters of the drop-casting method on electrodes’ electrochemical performance. Polymer–particle interactions proved to be crucial with regard to suspension homogeneity as well as quality of the deposited film. For positively charged copper sulfides, their steric stabilization with nonionic PVP resulted in well-dispersed particles and also ensured uniformity of the casted material layer. Electrosteric stabilization using anionic Nafion was efficient for suspension homogenization; however, it constricted flow pattern of particles leading to their concentration within the substrate center. For chitosan-based solutions, formation of aggregates was observed and dry deposits possessed characteristic ring of particles on the perimeter. In this research adjustment of drying conditions, which could additionally help in avoiding the coffee-ring effect, was not taken into consideration. Further investigation needs to be conducted in this direction. Moreover, the issue of film cracking observed for undiluted Nafion suspension (5 wt.%) should be addressed.

Drawing conclusions about materials functionality for glucose detection using electrodes prepared by the drop-casting method needs to be carefully executed. Various factors can influence an obtained signal, thus making any comparison between proposed nanostructures futile. This research aimed to underline the effect of applied binder for active material immobilization on sensor amperometric response. Conscious adjustment of a casted suspension properties could aid in obtaining more representative results. However, still fundamental studies are lacking, which would allow the design of materials on the stage of synthesis for specific sensing applications.

## Figures and Tables

**Table 1:** Composition of nanosuspensions.

Sample	Polymer	Polymer Content [wt.%]	Solvent	Copper Sulfides Content [wt.%]
CH_0.1/25%	Chitosan	0.1	25% CH_3_COOH	0.25
CH_0.5/25%	Chitosan	0.5	25% CH_3_COOH	0.25
CH_1.0/25%	Chitosan	1.0	25% CH_3_COOH	0.25
CH_0.5/1%	Chitosan	0.5	1% CH_3_COOH	0.25
CH_0.5/5%	Chitosan	0.5	5% CH_3_COOH	0.25
PVP_1.0/25%	PVP	1.0	25% CH_3_COOH	0.25
NAF_0.5	Nafion	0.5	mixture of isopropanol, n-propanol (ratio 11:9) and water (91.5–92.0%)	0.25

**Table 2:** Properties of the polymer solutions and nanosuspensions.

Sample	Polymer Solution		Nanosuspension
	Viscosity, *η* [mPa∙s]	pH	Hydrodynamic Diameter of Copper Sulfides, *D_hyd_* [µm]
CH_0.5/1%	14.36	2.84	4.04 ^1^
CH_0.5/5%	16.20	2.29	2.37 ^1^
CH_0.5/25%	21.37	1.85	2.02 ^1^
CH_0.1/25%	4.85	1.85	2.26 ^1^
CH_1.0/25%	73.12	1.85	4.20 ^1^
NAF_0.5	1.26	2.60	1.60 ^1^
PVP_1.0/25%	2.03	1.79	1.19 ^1^

^1^ Accuracy of the measurement depends on the material and material preparation procedure.

**Table 3:** Summary of electrodes electrochemical performance.

Sample	Peak Current, I (0.1 mM Glucose) [µA]	Oxidation Potential, Eo [mV]	LOD [mM]	LOQ [mM]	Active Surface Area, A [mm^2^]	Sensitivity, S [µA∙mM^−1^∙cm^−2^]
CH_0.5/25%	1.17 ± 0.21	540	0.008	0.024	1.79	655.29
NAF_0.5	2.42 ± 0.94	520	0.014	0.043	2.82	858.59
PVP_1.0/25%	6.23 ± 0.35	520	0.002	0.005	8.63	721.59

## Supplementary Materials

The following are available online at https://www.mdpi.com/1424-8220/21/3/802/s1, Figure S1: Volume distribution of copper sulfides particles in different solutions: water, CH_0.5/1%, CH_0.5/5%, CH_0.5/25%, CH_0.1/25%, CH_1.0/25%, PVP_1.0/25% and NAF_0.5. Figure S2: Microscope images of dry deposit of undiluted Nafion-based suspension (5 wt.% of polymer). Figure S3: Amperometric responses of two independent electrodes to glucose additions in the concentration range 0−1.0 mM (step 0.1 mM) for: (a) CH_0.5/25%; (b) NAF_0.5; (c) PVP_1.0/25%. Figure S4: Cyclic voltammograms for 1.5 mM glucose addition and their first derivatives for: (a) CH_0.5/25%; (b) NAF_0.5; (c) PVP_0.1/25%. Figure S5: Voltammograms recorded in 0.1 M KCl + 1 mM K_3_[Fe(CN)_6_] with different scan rates (6.25, 12.5, 25, 50, 100, 200, 250 and 500 mV/s) for: (a) CH_0.5/25%; (b) NAF_0.5; (c) PVP_1.0/25%. Figure S6: Amperometric responses to glucose in the concentration range 0−6 mM for CH_0.5/25%, NAF_0.5 and PVP_1.0/25%. Inset shows range from 0−0.1 mM. Figure S7: Raman spectra of pure polymers, copper sulfides, and casted nanosuspensions before and after CV measurements for: (a) CH_0.5/25%; (b) NAF_0.5; (c) PVP_1.0/25%.
