# Supplementary material for: Nonenzymatic Glucose Sensors Based on Copper Sulfides: Effect of Binder-Particles Interactions in Drop-Casted Suspensions on Electrodes Electrochemical Performance
Source: Sensors (Basel). 2021 Jan 26;21(3):802. doi: 10.3390/s21030802 (PMC7865286; doi:10.3390/s21030802)
Supplement: Supplementary file 1 [file sensors-21-00802-s001.pdf]

*Supplementary Materials*

# Nonezymatic Glucose Sensors Based on Copper Sulfides: Effect of Binder-Particles Interactions in Drop-Casted Suspensions on Electrodes Electrochemical Performance

**Julia Mazurków *, Anna Kusior and Marta Radecka**

Faculty of Materials Science and Ceramics, AGH University of Science and Technology, al. Mickiewicza 30, 30-059 Kraków, Poland; akusior@agh.edu.pl (A.K.); radecka@agh.edu.pl (M.R.)

* Correspondence: mazurkow@agh.edu.pl; Tel.: +48-12-617-24-68

**Number of Pages: 4**

**Number of Figures: 7**

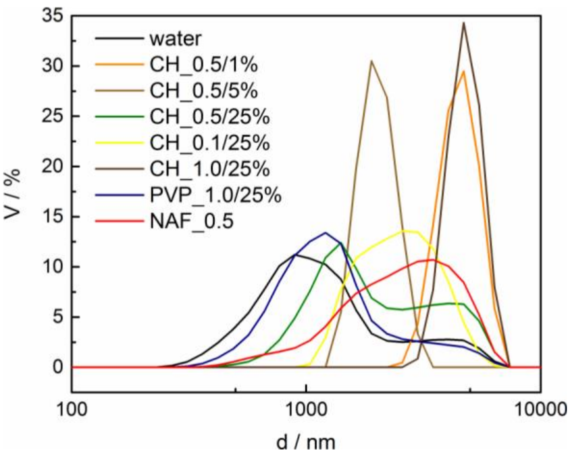


**Figure S1.** Volume distribution of copper sulfides particles in different solutions: water, CH_0.5/1%, CH_0.5/5%, CH_0.5/25%, CH_0.1/25%, CH_1.0/25%, PVP_1.0/25% and NAF_0.5.

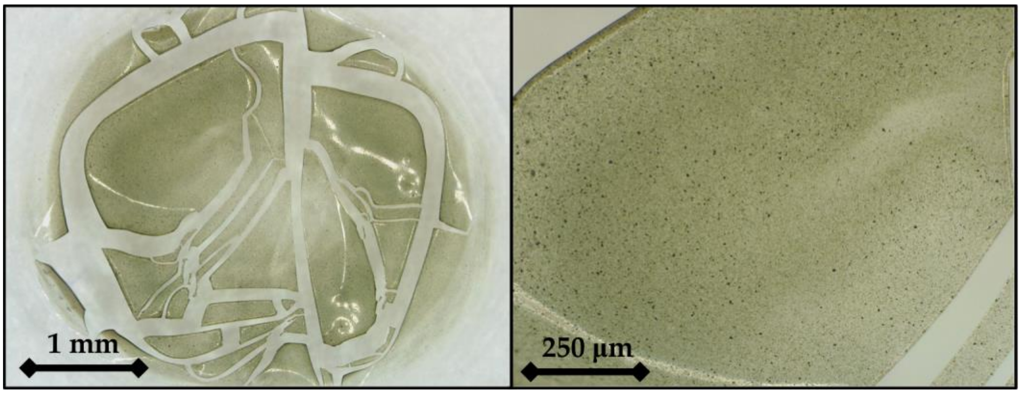


**Figure S2.** Microscope images of dry deposit of undiluted Nafion-based suspension (5 wt.% of polymer).

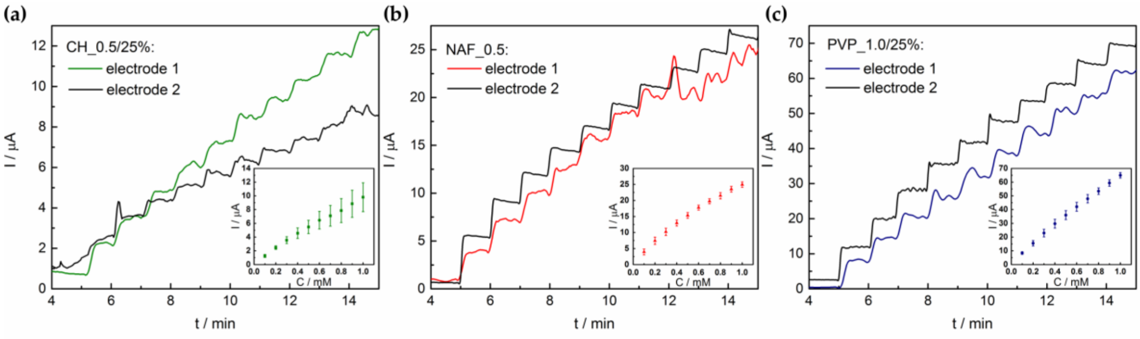


**Figure S3.** Amperometric responses of two independent electrodes to glucose additions in the concentration range 0–1.0 mM (step 0.1 mM) for: (a) CH_0.5/25%; (b) NAF_0.5; (c) PVP_1.0/25%.

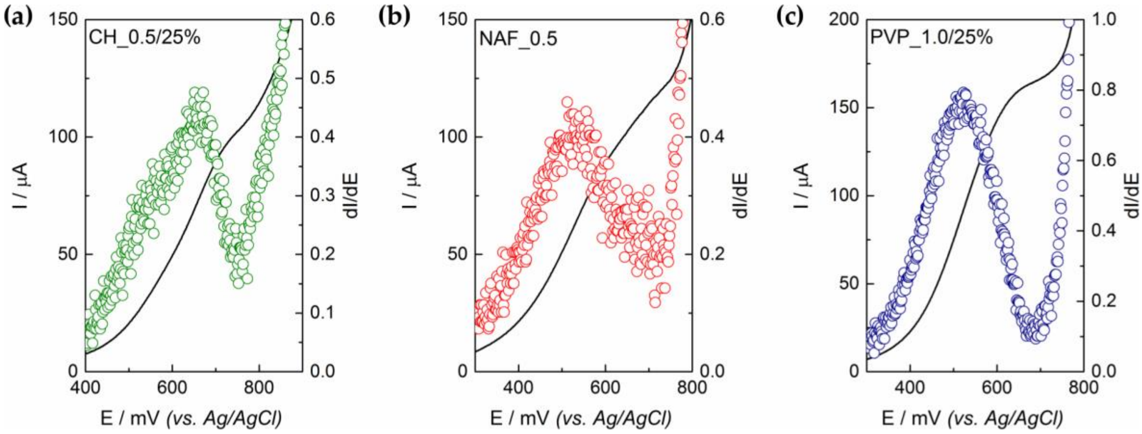


**Figure S4.** Cyclic voltammograms for 1.5 mM glucose addition and their first derivatives for: (a) CH_0.5/25%; (b) NAF_0.5; (c) PVP_0.1/25%.

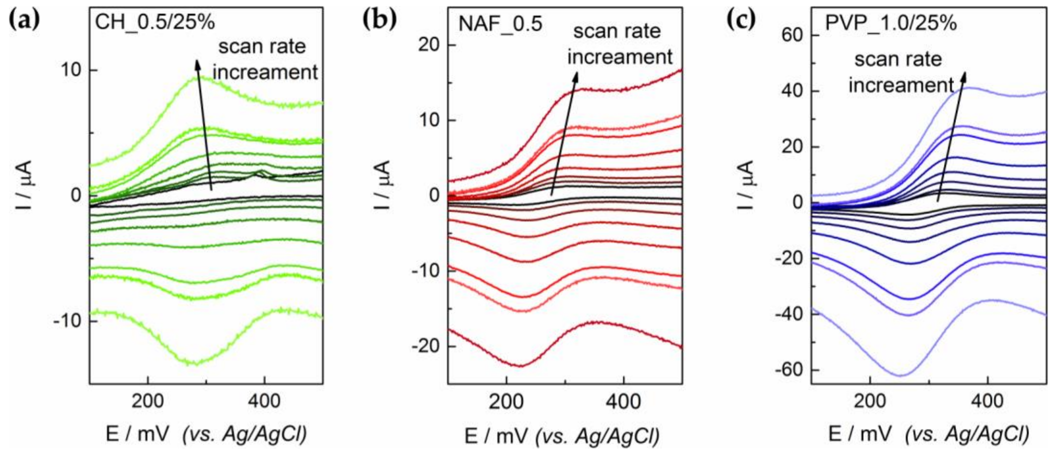


**Figure S5.** Voltammograms recorded in 0.1 M KCl + 1 mM $K_3[Fe(CN)_6]$ with different scan rates (6.25, 12.5, 25, 50, 100, 200, 250 and 500 mV/s) for: (a) CH_0.5/25%; (b) NAF_0.5; (c) PVP_1.0/25%.

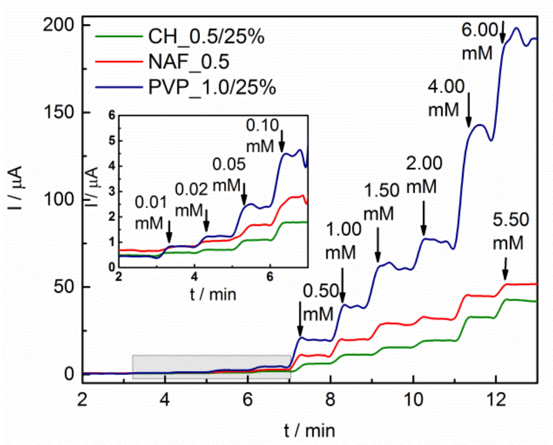


**Figure S6.** Amperometric responses to glucose in the concentration range 0−6 mM for CH_0.5/25%, NAF_0.5 and PVP_1.0/25%. Inset shows range from 0−0.1 mM.

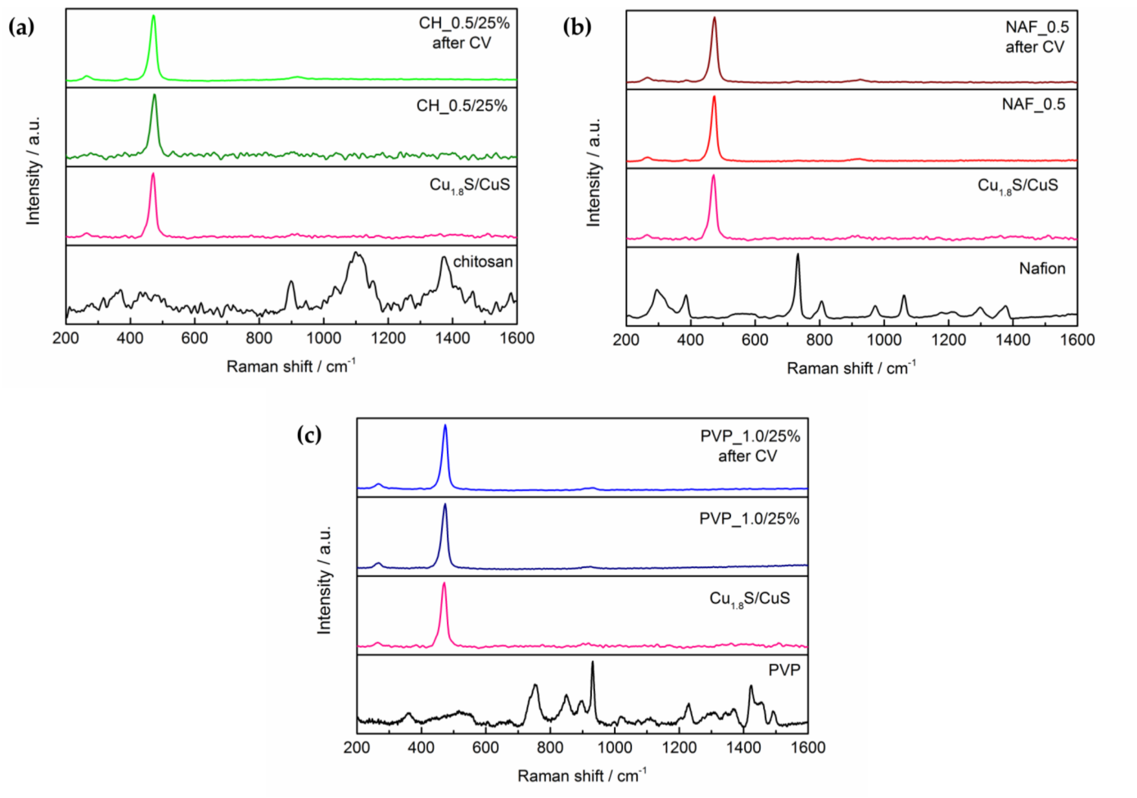


**Figure S7.** Raman spectra of pure polymers, copper sulfides, and casted nanosuspensions before and after CV measurements for: (a) CH_0.5/25%; (b) NAF_0.5; (c) PVP_1.0/25%.
